# Supplementary material for: In silico secretome analyses of the polyphagous root-knot nematode Meloidogyne javanica: a resource for studying M. javanica secreted proteins
Source: BMC Genomics. 2023 Jun 1;24:296. doi: 10.1186/s12864-023-09366-6 (PMC10236835; doi:10.1186/s12864-023-09366-6)
Supplement: Supplementary file 1 — Supplementary Material 1 [file 12864_2023_9366_MOESM1_ESM.docx]

**Additional file 1**

# *In silico* secretome analyses of the polyphagous root-knot nematode *Meloidogyne javanica*: A resource for studying *M. javanica* secreted proteins.


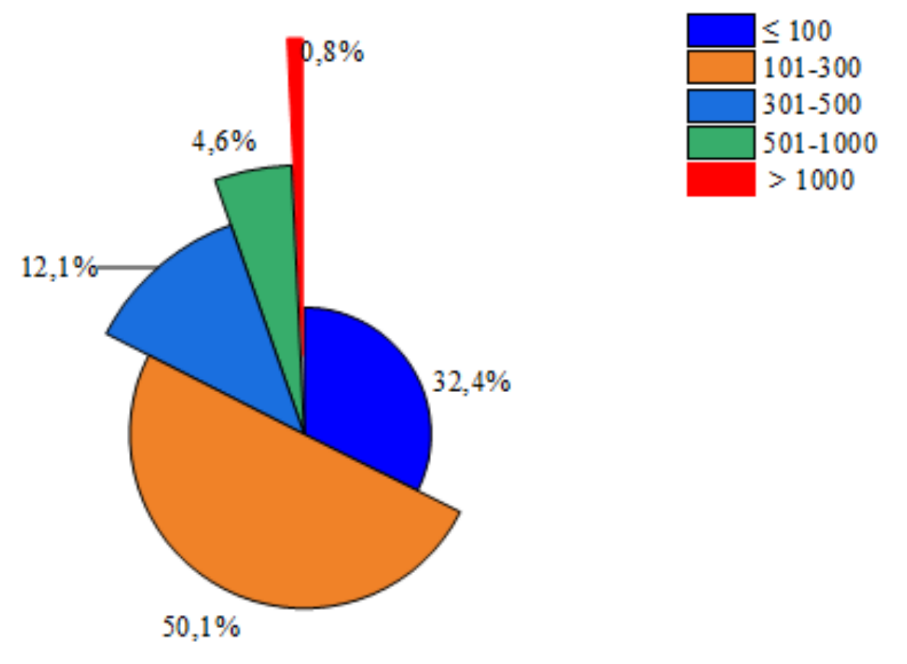


**Figure S1.** Proteins size distribution analysis in the predicted secretome of *M. javanica.*


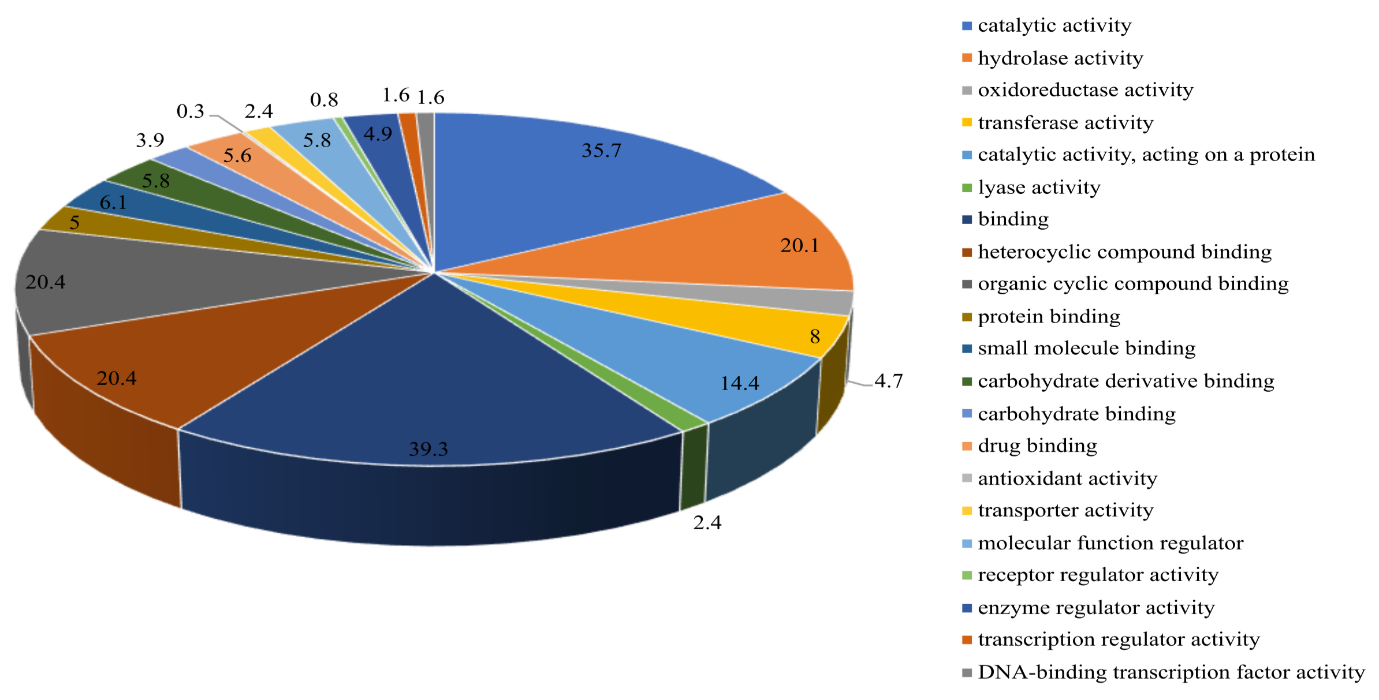


**Figure S2.** Distribution of Molecular function sub-categories of the predicted *M. javanica* secreted proteins.


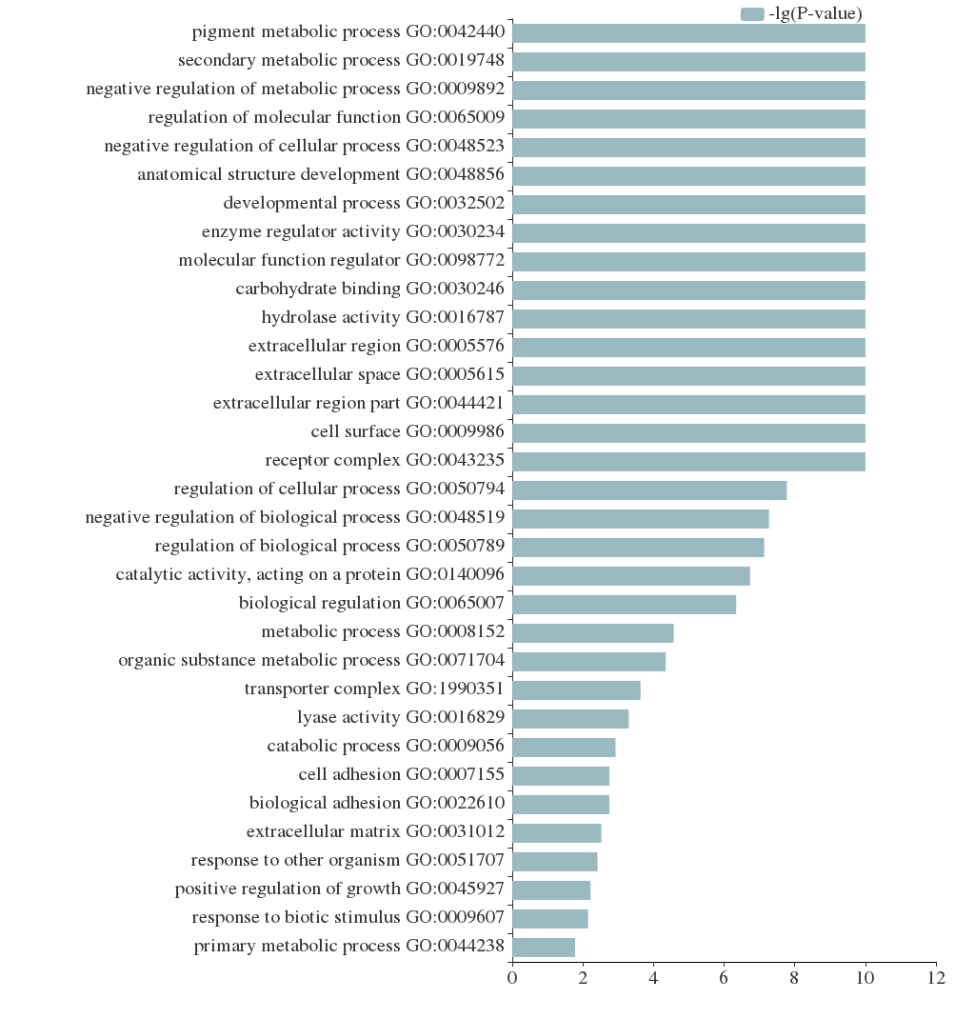


**Figure S3.** Gene ontology enrichment analysis of the predicted *M. javanica* secretome.

**Figure S4.** The distribution of cell-wall degrading enzymes functional classes in the predicted secretome of *M. javanica*.


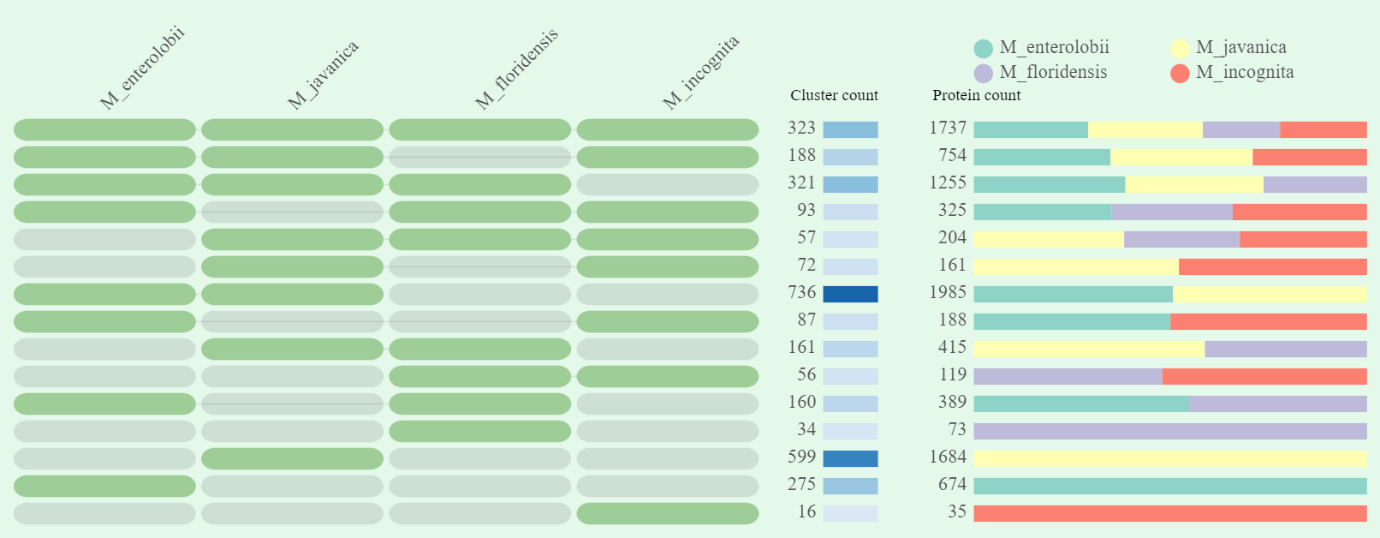


**Figure S5.** Orthovenn analysis illustrating the distribution of orthologous protein clusters of the predicted secretomes of four closely related *Meloidogyne* species. The unique and shared protein counts and clusters are shown.


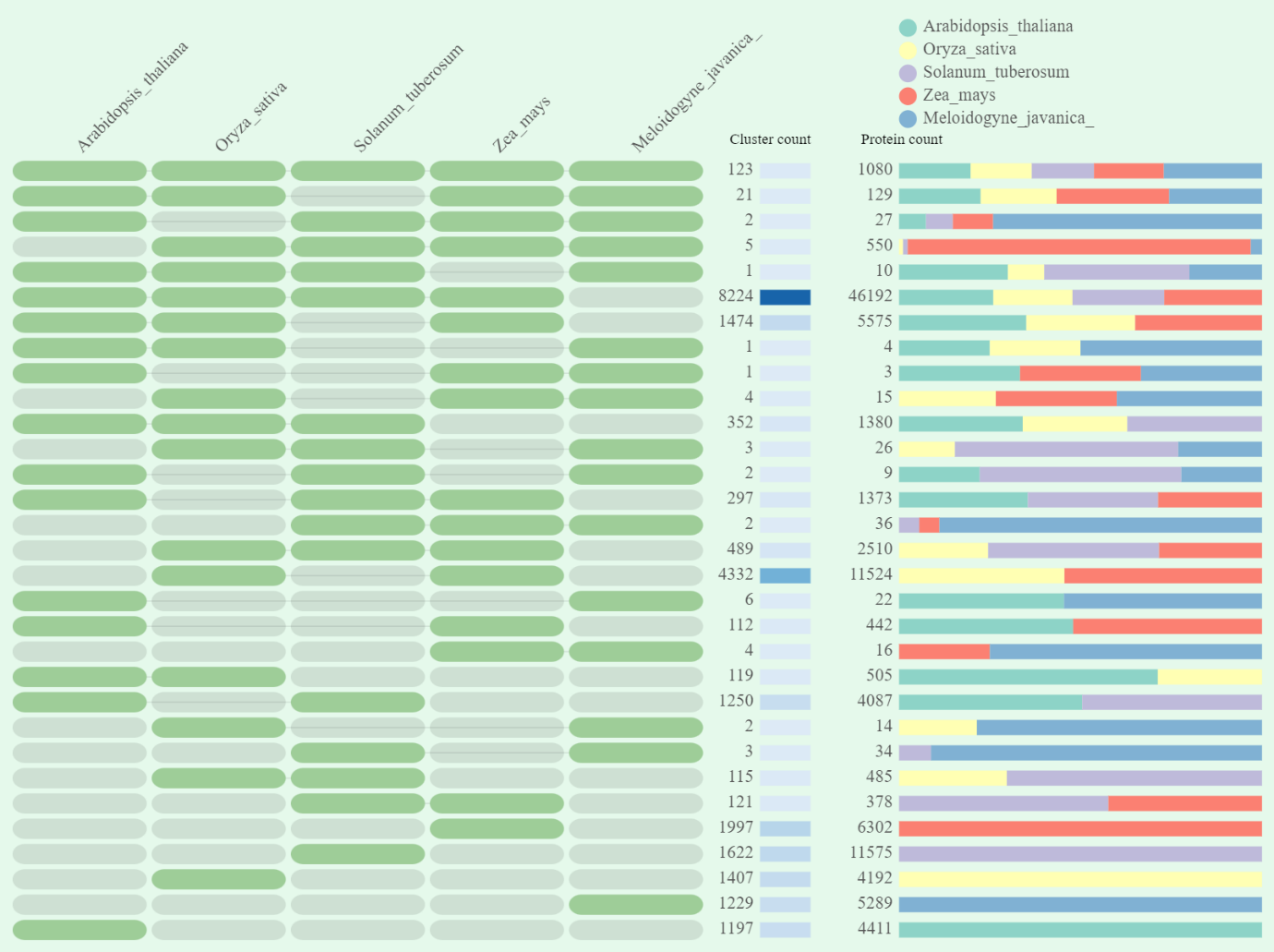


**Figure S6.** Cluster analysis depicting protein and cluster counts shared between *M. javanica* and host plant species.
